# L1_2_ ordering and *δ*′ precipitation in Al-Cu-Li

**DOI:** 10.1038/s41598-017-03203-z

**Published:** 2017-06-12

**Authors:** Pascal Neibecker, Michael Leitner, Muna Kushaim, Torben Boll, Dalaver Anjum, Tala’at Al-Kassab, Ferdinand Haider

**Affiliations:** 1 Heinz Maier-Leibnitz Zentrum (MLZ), Technische Universität München, Lichtenbergstr. 1, 85748 Garching, Germany; 2 King Abdullah University of Science and Technology (KAUST), 23955-6900 Thuwal, Kingdom of Saudi Arabia; 3 University of Augsburg, Department of Physics, Universitätsstr. 1, 86159 Augsburg, Germany; 4 Taibah University, Faculty of Science, Department of Physics, PO Box 344, Al-Madinah, Kingdom of Saudi Arabia; 5 Karlsruher Institut für Technologie (KIT), Institut für Angewandte Materialien - Werkstoffkunde (IAM-WK), Hermann-von-Helmholtz-Platz 1, 76344 Eggenstein-Leopoldshafen, Germany; 6 King Abdullah University of Science and Technology (KAUST), Imaging and Characterization Lab, 23955-6900 Thuwal, Kingdom of Saudi Arabia

## Abstract

The precipitation mechanism of the *δ*′ (Al_3_Li) phase in Al-Li alloys has been controversially discussed in recent decades, specifically with respect to a conjectured congruent ordering process. However, kinetics in the Al-Li system does not allow to resolve the intermediate stages of precipitation and hence to experimentally clarify this issue. In this paper, we are revisiting the subject in ternary Al-Cu-Li alloys with pronouncedly slower kinetics, employing Transmission Electron Microscopy, High-Angle Annular Dark-Field Scanning Transmission Electron Microscopy, Differential Scanning Calorimetry and Atom Probe Tomography. The results show clear evidence for congruent ordering in a selected compositional range, revealing an already strongly L1_2_ ordered microstructure after natural aging with a chemically homogeneous Li distribution and a decomposition of the alloy upon annealing at elevated temperatures. The presented study of the *δ*′ precipitation evaluates the reaction pathway of this process and compares it to the predictions of the Bragg-Williams-Gorsky model with respect to decomposition and ordering in this alloy system.

## Introduction

For decades, the formation mechanism of the *δ*′ (Al_3_Li) phase in Al-Li alloys has been the subject of controversial discussion^1–4^, since this process is assumed to take a complex pathway in the parameter space of chemical composition and order parameter as a function of temperature and time. Khatchaturyan *et al*.^3^ first parametrized a Bragg-Williams-Gorsky (BWG) model to fit the metastable *α*-*δ*′-miscibility gap. Analyzing the thermodynamics of this model, an interesting pathway, namely the spontaneous L1_2_-ordering of the matrix at room temperature followed by chemical decomposition during annealing at elevated temperatures, was conjectured.

Finding conclusive experimental evidence for this reaction pathway has however proven difficult in binary Al-Li alloys. As pointed out by Hono *et al*.^4^, it is impossible to ultimately evaluate the correctness of the proposed congruent ordering reaction since, due to fast kinetics, the entire reaction is already completed during quenching of a specimen. This claim was further confirmed in Refs 5–9, where *δ*′ precipitates were already found in the as-quenched samples. Only indirect evidence, as for instance investigations on the kinetics of the process by Schmitz *et al*.^10^, where the early stages of the precipitation process were quantitatively described by a modified Langer-Schwarz model, pointed towards a congruent ordering pathway. On the other hand, theoretical investigations by Abinandanan *et al*.^11^ employing Monte Carlo simulations could not find any evidence for congruent ordering in supersaturated fcc systems, questioning the validity of the mean-field BWG approach for modeling kinetics in general. Simultaneously, Small Angle X-ray Scattering experiments in combination with Wide Angle X-ray Scattering or calorimetric measurements performed on binary Al-Li alloys^1, 2, 12^ suggested that both processes, ordering and decomposition, happen concurrently but with different velocities. Recently, Kobayashi *et al*.^13^ reported evidence for a congruent ordering process in binary Al-11.7 at.% Li alloys employing high resolution TEM. Yet, the amount of contradicting studies on the precipitation mechanisms in binary Al-Li compounds casts doubt on the question whether in fact all parameters responsible for the precipitation processes are always in the experimenters’ control. This might especially be stressed in the light of new insight into the role of minor additions of supplemental elements such as Sn on the vacancy diffusion kinetics in Al alloys^14^.

The discovery of highly effective strengthening phases such as the T1 (Al_2_CuLi) phase in ternary Al-Cu-Li alloys have triggered profound interest in more complex precipitation-hardening Al alloys. Especially the Al-Cu-Li family shows here a desirable combination of mechanical properties such as a high Young’s modulus, a low density and a high yield strength^15, 16^. Besides these technical benefits, Al-Cu-Li alloys apparently show altered kinetics for Li diffusion, as Yoshimura *et al*.^17^ demonstrated a retardation of precipitation compared to binary Al-Li alloys. With Li diffusion being slowed down, the difficulties in discriminating between subsequent steps in the precipitation pathway of the *δ*′ phase can be evaded, making the Al-Cu-Li alloy family a suited model material for studying this process. Yoshimura *et al*.^17^ employed in their investigation High Resolution Transmission Electron Microscopy (HRTEM) and High-Angle Annular Dark-Field Scanning Transmission Electron Microscopy (HAADF-STEM) and found first evidence for congruent ordering in an alloy with 8.9 at.% Li and 1.3 at.% Cu but no such occurrence in an alloy with 6.1 at.%Li and 1.3 at.% Cu. While TEM is a very powerful technique for investigating (spatially resolved) states of order, drawing conclusions on chemical decomposition is challenging. Atom Probe Tomography (APT), giving access to 3D compositional information, has successfully been employed in resolving the early stages of precipitation in Al alloys^18–21^, and its combination with TEM seems the optimal set of experimental techniques to contribute to the open congruent order debate.

## Results

### The Al-Li metastable phase diagram

**Figure 1:**
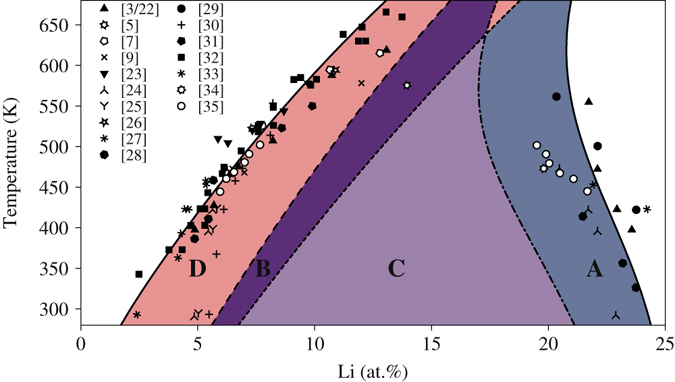
Phase diagram of the pseudobinary Al(-Cu)-Li system as determined from the described BWG model parametrized using the experimental data sets reported in refs 3, 5, 7, 9, 22–35. A–D denote distinctive regions of thermodynamic ordering and decomposition stability as described in the text. Together they form the metastable miscibility gap between solid solution and L1_2_ phase. In the figure, open symbols refer to microscopy techniques, full symbols refer to bulk and spectroscopy techniques and line symbols refer to scattering techniques.

The metastable phase diagram of the pseudobinary Al-Li system can be calculated employing the Bragg-Williams-Gorsky (BWG) model, as firstly demonstrated for this system by Khatchaturyan *et al*.^3^ Yet, the plenitude of experimental data available today, especially for the Al-rich side of the miscibility gap, allows for a more accurate parameterization of the model than the single data set used in the original Khatchaturyan paper. Figure 1 shows the reparametrized metastable phase diagram of the pseudobinary Al-Li system calculated with the BWG model. A detailed description of the calculations is given in the Supplementary Information. The reparametrization is based on a comprehensive set of experimental data obtained with a variety of experimental methods such as DSC, Small Angle X-ray Scattering, Small Angle Neutron Scattering, Electrical Resistometry, Positron Annihilation Lifetime Spectroscopy, TEM and APT^3, 5, 7, 9, 22–35^. While most data sets refer to binary Al-Li alloys, others refer to ternary or even multicomponent technical alloys. Yet, considering the high conformity of the data sets with respect to the *δ*′ miscibility gap, all systems can be considered to be pseudobinary.

In the Al-Li system, the thermodynamics with respect to the fcc Al and the L1_2_ ordered *δ*′ (Al_3_Li) phases is more complicated than in simple phase-separating systems due to the additional freedom in the degree of order. Specifically, the region of phase separation splits into 4 distinct regions (Fig. 1): In all regions, a homogeneous disordered system as obtained by high-temperature quenching is unstable with respect to decomposition. The used nomenclature for the 4 regions (A–D) is the one introduced previously by Khatchaturyan *et al*.^3^.

The analysis shows that in region D, disorder is stable in the matrix and the ordered Li-rich phase has to be nucleated. In regions A and C, the disordered matrix is unstable with respect to spontaneous homogeneous ordering. The resulting ordered matrix is then unstable with respect to spinodal decomposition in region C, while in region A the disordered Li-lean phase has to be nucleated. Finally, in region B matrix disorder is metastable, but ordered regions would undergo spinodal decomposition, so that a priori it is unclear whether a system quenched into a given point in B would proceed by first nucleating order and then spinodally decomposing, or rather taking the direct way of nucleating Li-rich precipitates as in region D.

## Experimental Results

Here, we report on the early stages of precipitation upon aging at 433 K in the Al-Cu-Li model alloy Al- 1.0 at.% Cu- 5.9 at.% Li, starting with solution heat-treated, quenched and naturally aged samples. A combination of APT, TEM, HRTEM, HAADF-STEM and Differential Scanning Calorimetry (DSC) enables us to address the question of congruent order, the precipitation process of the *δ*′ phase as well as Cu-Li correlations at early stages of the decomposition. Furthermore, the collected data allows for a critical assessment of the BWG model with respect to its predictive power for the interplay between atomic order and chemical decomposition in the formation process of the *δ*′ phase. The detailed experimental procedures are outlined in the Methods section.

### Transmission Electron Microscopy (TEM)

**Figure 2:**
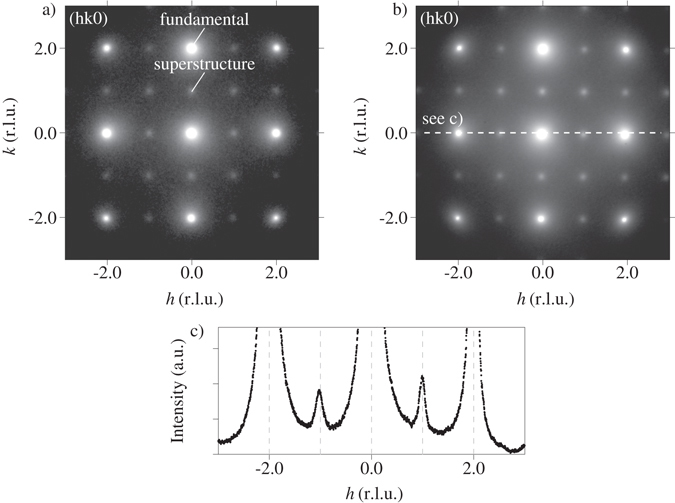
Electron diffraction image along the [001] direction of the Al-Cu-Li alloy in (**a**) naturally aged and (**b**) naturally aged plus 5 minutes at 433 K artificially aged condition. L1_2_ superstructure reflections are clearly visible in both aging conditions. (**c**) depicts a line intensity profile as indicated in (**b**).

Figure 2 shows electron diffraction patterns of the Al-Cu-Li alloy along the [001] direction for the naturally aged condition (Fig. 2a) and the naturally aged plus 5 minutes at 433 K artificially aged condition (Fig. 2b). Besides the Al fcc reflections, clear L1_2_ superstructure reflections can be observed in both aging conditions. Between the naturally aged and the naturally aged plus 5 minutes at 433 K artificially aged condition, no apparent differences are observed. Both the intensity and the widths of the superstructure reflections are similar.

From the widths of the superstructure reflections, estimates about the corresponding correlation length of the ordered domains can be retrieved. We determined the half width at half maximum (HWHM) Γ of the superstructure reflections via fitting cuts through 7 superstructure reflections with Lorentzian functions, giving Γ = 0.155 nm^−1^ (±0.006 nm^−1^). According to ref. 36, the correlation length *L* of the ordered domains can be calculated as *L* = 0.443/Γ. Hence, we determined the corresponding correlation length of the ordered domains as *L* ≥ 2.86 nm (±0.1 nm). This value is to be understood as a lower bound of the correlation length, taking into account that instrument resolution effects additionally broaden the reflections which we did not model explicitly. While the electron diffraction images indicate the presence of a L1_2_ phase with relatively large coherent domains, it should be emphasized that they give no information about the origin of the superstructure reflections, namely whether they are due to homogeneous matrix ordering or rather incipient decomposition with ordered precipitates in a disordered matrix.

**Figure 3:**
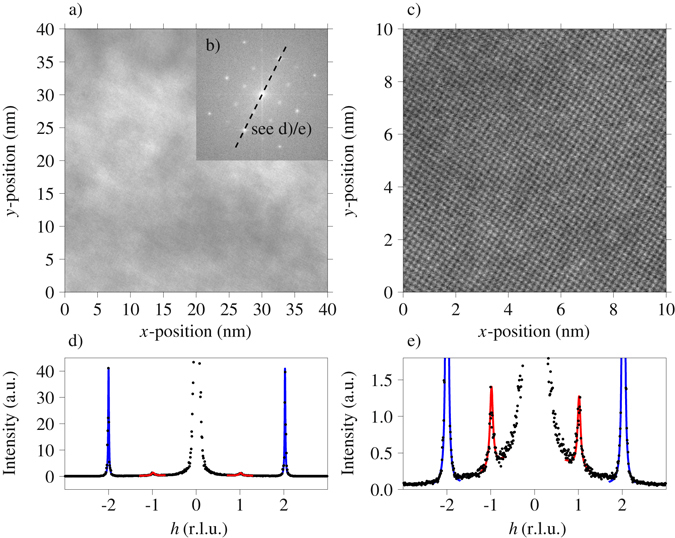
HRTEM image of the Al-Cu-Li alloy in the naturally aged condition (**a**) with its FFT given in (**b**) on a logarithmic scale. (**c**) shows a HAADF-STEM image of the sample. (**d**,**e**) give the intensity along the same line cut as indicated in (**b**) analogous to Fig. 2(c), where the *y*-scale in (**e**) is magnified in comparison to (**d**). In (**d**,**e**), the fundamental and superstructure reflections have been fitted with Lorentzian functions (blue and red lines). The HRTEM image in (**a**) has a linear gray scale with black corresponding to zero intensity. In the HAADF-STEM image (**c**) the dynamic range (black to white) corresponds to a variation in the detected intensity of 7 %.

To test for precipitation, HRTEM and HAADF-STEM investigations have been performed. Figure 3(a) depicts the (40 mm)^2^ HRTEM image of a sample in naturally aged condition. Figure 3(b) depicts the FFT of Fig. 3(a) on a logarithmic scale showing that the superstructure reflections observed in the electron diffraction images of Fig. 2 can be clearly resolved also in the FFT of the HRTEM images. A (10 nm)^2^ HAADF-STEM image is given in (c). Figure 3(d,e) show line intensity profiles as indicated in (b) in analogy to Fig. 2(c). The width of both the superstructure reflections and the fundamental reflections was determined via fitting with Lorentzian functions. As described above, the corresponding correlation length was calculated, yielding values of 17.7 nm for the fundamental reflections and 4.3 nm for the superstructure reflections. While the fundamental reflections have a correlation length limited essentially by the size of the analyzed region, the L1_2_ superstructure reflections are clearly broadened. As can be seen, the correlation length estimations from HRTEM and electron diffraction show reasonable agreement, with the increased width in electron diffraction being probably due to instrument resolution, which is much less critical for the FFT analysis of the HRTEM image.

Both HRTEM and HAADF-STEM show no evident precipitation. This suggests that the observed broadening of the superstructure peaks is rather due to homogeneous matrix ordering with small antiphase domains than to well-developed precipitates, while we cannot exclude early-stage precipitates with sizes below what can be resolved in real-space HRTEM and STEM images.

### Atom Probe Tomography (APT)

To test for the existence of nm-scale precipitates in connection to the observed superstructure in electron microscopy, APT measurements have been performed on samples in different heat-treatment conditions. Our APT data evaluation is based on a binomial distribution analysis that identifies compositional variations in small and compact analysis volumes comprising *M* reconstructed atoms. Details on the employed algorithm are given in the Supplementary Information.

**Figure 4:**
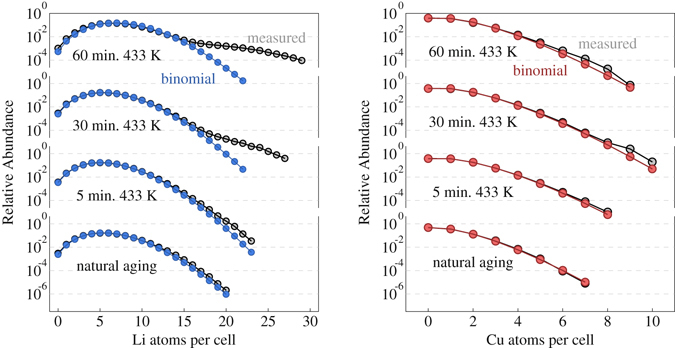
Li and Cu distribution analysis in naturally aged, 5 minutes at 433 K aged, 30 minutes at 433 K aged and 60 minutes at 433 K aged condition (cell size of 100 atoms).

Figure 4 depicts the experimentally obtained distribution of Li and Cu atoms, respectively, within analysis cell sizes of *M* = 100 together with the binomial distributions for the respective concentrations. While both distributions do not show large deviations from the ideal binomial distribution at short annealing times, starting with 30 minutes of artificial aging at 433 K the Li distribution clearly deviates from the binomial distribution, giving rise to a second maximum at larger Li concentrations around 22 at.% Li. For Cu, at the same time, only very minor deviations from the binomial distribution are observed even for larger annealing times, which indicates that there are no significant correlations of Cu atoms in the investigated annealing stages, neither in terms of explicit Cu clustering such as in Guinier-Preston zones nor via a correlation to the developing Li-rich precipitates. The latter observation corroborates our hypothesis of thermodynamic similarities between Al-Li and Al-Cu-Li alloys especially in early stages of precipitate formation.

**Figure 5:**
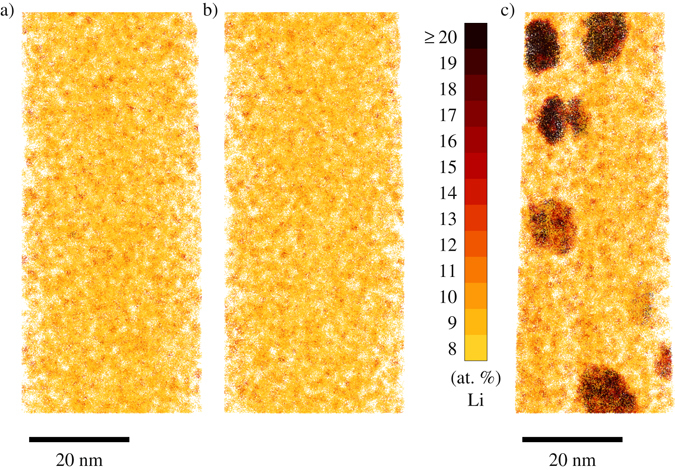
Visual representation of the spatial distribution of Li clusters for randomly labeled data (**a**), the experimentally observed distribution in the 5 minutes at 433 K artificially aged sample (**b**), and the experimentally observed distribution in the 60 minutes at 433 K artificially aged sample (**c**). The central atom of any 100 atom cell is given a color depending on the Li concentration in the respective cell. While bright yellow stands for analysis cells with 8 at.% Li, dark red indicates a Li composition of ≥20 at.% Li.

The concentration maps of the reconstructed samples are illustrated in Fig. 5. Panel (b) depicts the 5 minutes at 433 K artificially aged condition while panel (a) shows a random Li distribution on the reconstructed atomic positions of the same sample (commonly referred to as random labeling^37^ or random relabeling). The visual similarity confirms, on top of the statistical evidence discussed above, the absence of decomposition in the very early stages of aging. A larger APT reconstruction of the 5 minutes at 433 K artificially aged sample is additionally given in the Supplementary Information. Similar analyses for Cu confirm the above-stated absence of clusters in all investigated annealing conditions. Figure 5(c) shows the spatial distribution of Li in a progressed state of aging, specifically in the 60 minutes at 433 K artificially aged condition. Chemical decomposition is well pronounced in this aging condition, revealing the presence of spherical *δ*′ precipitates with an average radius of approximately 6.9 nm, a Li concentration in the core of a well developed precipitate of approximately 22.5 at.% Li and a precipitate volume fraction of approximately 0.72 % as averaged over 2 samples analyzed in this heat treatment condition and where all analysis cells with a Li concentration of ≥13 at.% were defined as representing the precipitate volume.

A major question in the understanding of current high strength Al alloys is the formation mechanism of crucial ternary phases such as for example the T1 phase (Al_2_CuLi) in Al-Cu-Li alloys. Having been subject to intensive investigation, it is believed today that additional alloying elements such as Ag and Mg facilitate the formation of the respective T1 phases^17^, while in pure ternary alloys its formation is impeded. The statistical information contained in the APT data with respect to mutual clustering tendencies allows now to evaluate correlations in the Li and Cu distribution in very early stages of precipitate formation. Cu-Li correlations are directly accessible via the two-dimensional local composition histograms generated by our data analysis algorithm. However, these histograms reveal no statistically significant deviation from randomness in the annealing stages studied here, indicating that there is no general Cu-Li clustering tendency in the system.

### Differential Scanning Calorimetry (DSC)

**Figure 6:**
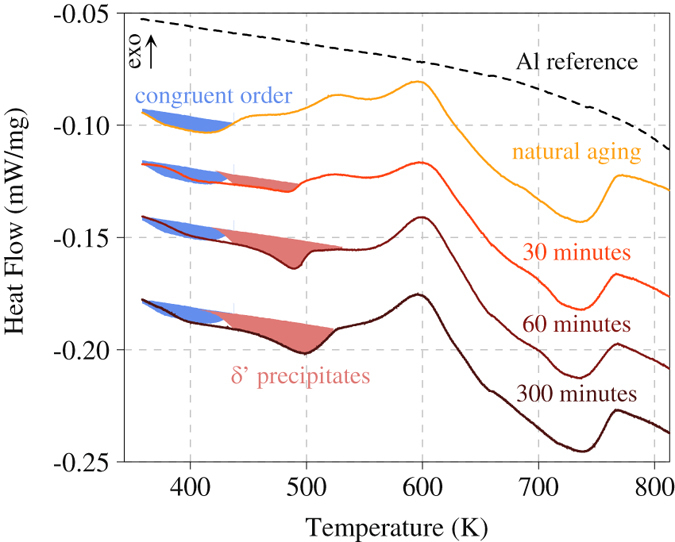
DSC measurements of the Al-Cu-Li model alloy in 4 heat treatment conditions (naturally aged as well as 30 minutes, 60 minutes and 300 minutes artificially aged at 433 K).

Finally, Fig. 6 shows DSC measurements of the Al-Cu-Li alloy in 4 different heat treatment conditions, namely naturally aged, as well as naturally aged plus 30 minutes, 60 minutes and 300 minutes artificially aged at 433 K. In the naturally aged condition, a first endothermic peak is observed at around 400 K with an onset temperature of approximately 360 K. Based on the TEM and APT results presented above, this first endothermic peak is assigned to the matrix disordering.

While this first peak seems to be present in all aging conditions (blue shaded area in Fig. 6), in the artificially aged samples a second endothermic peak at around 480 K is emerging with increased aging time, which can consequently be ascribed to the dissolution of *δ*′ precipitates (red shaded area in Fig. 6). This interpretation is supported by this second endothermic peak shifting to the right as well as increasing in area with increased aging time, as commonly observed when precipitate size as well as precipitate volume fraction grow. Since the first two endothermic peaks in the artificially aged samples are superimposed, the determination of the onset of the *δ*′ dissolution peak proves difficult. Yet, assuming the calorimetric signal of matrix disordering to stay relatively constant in shape and size throughout the different heat treatment conditions, an approximate value for the onset temperature of 430–440 K can be estimated.

The peaks at higher temperatures seem to be little affected by the applied heat treatment and presumably belong to *in-situ* formation and dissolution of phases such as the *δ* (AlLi), the *θ*′′ phase (Al_3_Cu), the *θ*′ phase (Al_2_Cu) and the stable *θ* phase (Al_2_Cu), while also the formation of ternary phases such as the T1 phase (Al_2_CuLi) are plausible but shall not be further discussed in the scope of this manuscript.

## Discussion

### Interpretation of the experimental results

The presented results draw a comprehensive picture of the precipitation pathway of the *δ*′ (Al_3_Li) phase in Al- 1.0 at.% Cu- 5.9 at.% Li. Apart from the significance for the Al-Cu-Li system itself, by analogy the presented results also constitute a strong evidence for the formation mechanism of the *δ*′ phase in Al-Li alloys. This is especially true in early stages of precipitate formation, where no apparent Cu-Li correlations are found. In fact, only the altered Li diffusion kinetics in the Cu containing alloys allows for the discrimination of early steps in the *δ*′ precipitation pathway usually obscured in binary Al-Li alloys.

In the naturally aged condition, TEM results show clear evidence of an L1_2_ superstructure, visible both in the direct electron diffraction image (Fig. 2) as well as in the Fourier transform of the HRTEM image (Fig. 3). The question is now whether this is due to homogeneous matrix ordering or rather due to ordered Li-rich precipitates in a disordered matrix. Our APT results of the naturally aged as well as 5 minutes artificially aged condition show only minor deviations from a binomial Li distribution in 100 atoms analysis cells, indicating that there is no pronounced decomposition on the length scale of these cells. With an approximate detection efficiency of 30 % in APT, the approximate radius of a 100 atoms analysis cell is 1 nm. On the other hand, an estimation of the correlation length corresponding to the superstructure reflections observed in TEM yields values in the order of 3–4 nm. Of course, the correlation length of long-range order in ordered precipitates cannot be larger than the precipitate size, thus necessitating an ordered matrix to explain the situation. To further corroborate this statement, for the early stages of precipitation smaller analysis cell sizes have additionally been investigated, confirming a quasi chemically homogeneous Li distribution in the naturally aged and also in the 5 minutes artificially aged condition. Yet, considering a resolution of the atom probe of 0.5 nm in *x*-*y* direction as well as the limited detection efficiency raises the question whether much smaller analysis cells than 1 nm are in fact meaningful.

Using APT, subsequently the decomposition process upon artificial aging at 433 K was followed by means of a binomial distribution analysis coupled to a real space depiction of the retrieved elemental distributions (Figs. 4 and 5). In the case of Li, it is first of all evident that already in the early stages of heat treatment the measured Li distribution is slightly broader than the ideal binomial distribution. Yet, these deviations are so small that they cannot be ascribed to fully formed precipitates. Rather, they are presumably due to sub-critical short-range clustering fluctuations present in the sample, so that the occupations within an analysis cell are not strictly independent. Quantitatively, the resulting broadening with respect to the binomial distribution can be modelled by a Markov chain, with the Li-Li pair probability increased over the uncorrelated probability by about 20 %.

Obvious Li-rich precipitation is first found in samples aged for 30 minutes at 433 K, where a few but well-defined precipitates are present (see Supplementary Information). In the local composition histograms, these precipitates lead to a second component in the spectrum with Li concentrations around 22at.% as shown in Fig. 4, while at medium Li concentrations the deviation from the ideal binomial distribution is minor. These observations point towards a nucleation and growth mechanism of precipitate formation at the annealing temperature of 433 K. A formation process relying on spinodal decomposition can almost certainly be excluded, since in no state a homogeneous broadening of the Li distribution curve is observed in the local composition histograms. In the case of Cu, in all investigated annealing stages no significant deviations from randomness are found.

Employing DSC, both the endothermic disordering signal of the L1_2_ ordered matrix phase as well as, in later heat-treatment stages, the endothermic dissolution peak of existing *δ*′ precipitates could reliably and independently be followed through the different heat-treatment conditions. The presence of the matrix disordering signal in all heat-treatment conditions (Fig. 6) suggests that the matrix composition upon formation of *δ*′ precipitates remains above the threshold value for order stability at room temperature. Consequently, in all heat-treatment conditions, the matrix returns upon cooling to room temperature into a L1_2_ ordered state. Annealing at 433 K leads to the formation of *δ*′ precipitates that grow in size and volume fraction with increased annealing times. The impeded formation of precipitates at room temperature is thereby not of thermodynamic but of kinetic reasons, taking into consideration that the adjustment of order as a very local process relies only on a few jumps per atom, while precipitate formation relies on long-range atomic diffusion processes effective only at higher temperatures.

The absence of correlations in the Li and Cu distribution in the investigated early stages of aging indicate that Li and Cu do not influence each other initially, besides the fact that the diffusion kinetics for Li is apparently reduced in ternary Al-Cu-Li alloys.

In principle, the described findings are in good agreement to the observations reported by Yoshimura *et al*.^17^ in an alloy with comparable Li and higher Cu content. Admittedly, Yoshimura *et al*. point out that the transition from region D in the pseudobinary Al-Li phase diagram (Fig. 1) where disorder is stable in the matrix and region B where matrix disorder is metastable has to occur above 6.1 at.% Li, while in this study the matrix order was still found in an alloy with 5.9 at.% Li. Nonetheless, the small deviations in Li composition of the alloys in the two studies together with the inherent errors in the determination of Li contents in general can plausibly account for this inconsistency. Apart from this, the absence of *δ*′ precipitates in the naturally aged and 180 minutes at 373 K annealed microstructure and their appearance after annealing at elevated temperatures of 473 K agree well with our experimental observations. The absence of GP zones in the alloy studied in this work in comparison to the alloy studied by Yoshimura *et al*. is most likely caused by the smaller Cu content in our alloy.

### Predictions of the Bragg-Williams-Gorsky (BWG) model

The BWG model corresponding to the phase diagram in Fig. 1 allows to assess the pathway of ordering and decomposition at various compositions and temperatures. For the composition investigated in this study, the distinct thermodynamic situations projected by the BWG model at room temperature (293 K), solution annealing temperature (803 K) and artificial annealing temperature (433 K) will be outlined.

**Figure 7:**
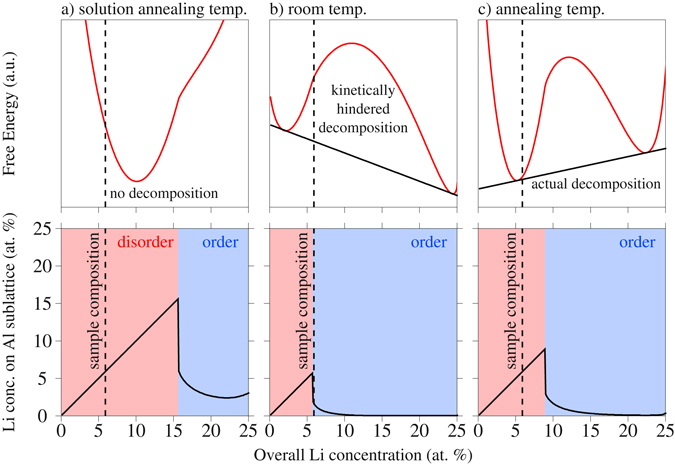
Free energy curves according to the BWG model at three different temperatures, namely solution treatment temperature (**a**), room temperature (**b**) and annealing temperature (**c**). The upper part of the figure shows the calculated free energy curves for fixed homogeneous composition, but free degree of long-range order, while the lower part illustrates the corresponding long-range order parameter via the Li concentration on the Al sublattices.

Figure 7 shows free energy curves calculated for the 3 temperatures (upper part of Fig. 7) together with the state of order at the respective temperatures and concentrations via the Li occupation on the Al sublattices (lower part of Fig. 7; *c*_2_ in the scope of the terminology introduced in the Supplementary Information). The dashed line represents the Li concentration of the alloy investigated in this study. It should be noted that the state of order at any composition is in fact a free parameter in the BWG model and our representation here is only the minimum of the free energy curves at any temperature and composition with respect to the state of order. Thereby we consciously omit questions of the (meta)stability of disorder, an issue specifically discussed in the original work of Khachaturyan^3^, yet probably almost impossible to observe experimentally.

At the solution treatment temperature (Fig. 7a), according to the BWG model a disordered solid solution is stable. At room temperature (Fig. 7b), the BWG model predicts an ordered and chemically unstable state that would demix via a spinodal decomposition process into a Li-lean matrix and Li-rich precipitates. Yet, as discussed above, presumably the limited kinetics at room temperature in reality impede this decomposition process. At the artificial annealing temperature (Fig. 7c), the homogeneous state is predicted to be disordered and unstable with respect to decomposition, which will occur via a nucleation and growth mechanism.

**Figure 8:**
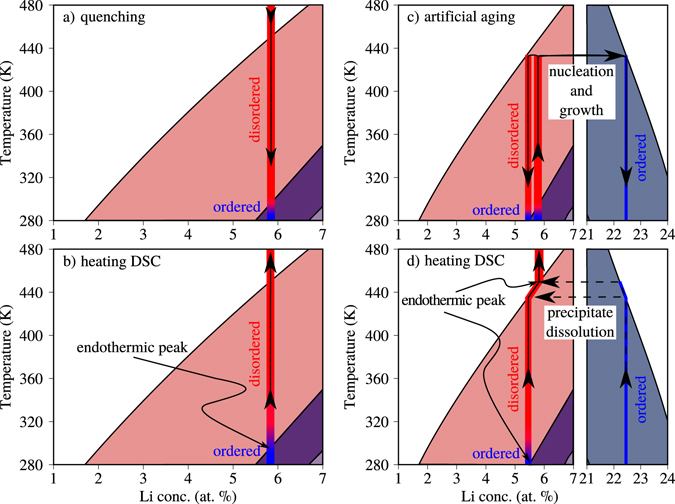
Reaction pathway of the sample during quenching from solution annealing temperature (**a**) and during subsequent slow heating from room temperature as performed in the DSC (**b**), as well as during annealing at 433 K with subsequent quenching to room temperature (**c**) and during subsequent slow heating from room temperature as performed in the DSC (**d**).

The predictions of the BWG model are compared in Fig. 8 to the experimental observations presented in the scope of this study. The projected reaction pathway is, in order to illustrate the processes, drawn into the BWG based phase diagram. Specifically, Fig. 8(a) presents the pathway taken by the sample upon quenching from the solution treatment temperature and subsequent natural aging. First of all, the chemically homogeneous alloy experiences a change from order instability at solution treatment temperature to order stability at room temperature. The quenching preserves the high temperature homogeneous state to room temperature by bypassing the medium temperature regions where atomic diffusion is fast enough to accommodate decomposition processes. During subsequent natural aging, in principle there are thermodynamic driving forces for two processes, the adjustment of long-range L1_2_ order and the spinodal decomposition of the matrix. However, the atomic mobility at room temperature is only sufficient to accommodate a change in the state of order, while the spinodal decomposition process does not get past its initial stages that can hardly be observed experimentally. This scenario is in good agreement with the presented experimental results, where a strong L1_2_ order is observed but no pronounced chemical decomposition is found in the as quenched and naturally aged samples.

Figure 8(b) depicts the situation during a DSC measurement of the as quenched and naturally aged sample. Here, upon slow heating, the disappearance of the L1_2_ order above 300 K is projected. By comparison, the presented DSC measurements indeed reveal an endothermic signal that can be ascribed to such matrix disordering with a slightly higher onset temperature of approximately 360 K.

Figure 8(c) denotes the thermodynamic situation during artificial aging of the naturally aged samples. First, during heating to the aging temperature, the ordered matrix is expected to disorder at approximately 300 K. At the aging temperature of 433 K, then, a decomposition reaction via a nucleation and growth process is projected, meaning that *δ*′ precipitates with a composition of approximately 22 at.% Li will be nucleated and grow subsequently. Such a nucleation and growth process is indeed well resolved in the statistical analysis of the Li distribution in the APT measurements. The nucleation of the precipitate phase is clearly observed and the retrieved Li concentration in the center of the precipitates of 22.5 at.% agrees well with the prediction of the BWG model. Quenching the specimens after artificial aging in the demixed state to room temperature is predicted to result in a Li-depleted matrix with approximately 5.5 at.% Li. In the presented phase diagram, the matrix composition at room temperature that still shows stability of the ordered phase is just around these expected matrix compositions after decomposition at 433 K. As discussed above, the stability of the first disordering signal in the DSC measurements over all annealing conditions is a strong argument for the stability of order even in the Li-depleted matrix after artificial aging.

Figure 8(d) depicts the situation during subsequent measurement of the artificially aged samples in a DSC experiment. According to the BWG phase diagram, a primary endothermic peak corresponding to matrix disordering would be expected, if at all present, at approximately 250 K, while a second endothermic peak corresponding to the dissolution of the precipitate phase should appear at approximately 440 K. In the actual experiment, we observe the onset of the primary disordering signal at slightly higher temperatures of 360 K and the *δ*′ dissolution peak, in good agreement to the BWG model, at around around 430–440 K.

In general, quantitative predictions of a BWG model have to be taken with care. Here, the model is parametrized in order to fit the experimentally known phase boundaries, and the phase space of metastable matrix order is derived from this parametrization. Effects of correlation, which are disregarded in the BWG model, will likely shift these boundaries with respect to each other. Still, we observe a good qualitative agreement of thermodynamic situations along the reaction pathway between our experimental results and the BWG predictions as detailed above.

## Conclusion

In this paper, we discussed the precipitation mechanism of the *δ*′ phase in Al-Cu-Li alloys, specifically in Al- 1.0 at.% Cu- 5.9 at.% Li, based on experimental investigations by TEM, APT and DSC. We made use of the fact that the ternary system, in comparison to binary Al-Li alloys, allows kinetically for a clear resolution of the various steps of the reaction pathway. It was found that upon quenching from the solution treatment temperature with subsequent natural aging, Li first orders homogeneously, while neither for Li nor Cu a chemical decomposition was detected in this heat-treatment condition. As evidenced by statistical analysis of the APT results, during subsequent artificial aging at 433 K, the matrix demixes in a nucleation and growth process. Eventually this results in a matrix with a composition of approximately 5.5 at.% Li, which is ordered at room temperature, and *δ*′ precipitates of an average diameter of 6.9 nm after an annealing treatment of 60 minutes. The experimental observations qualitatively perfectly confirm the predictions of the Bragg-Williams-Gorsky (BWG) model with respect to the interplay of ordering and decomposition processes and our results give strong evidence for the correctness of the projected congruent ordering mechanism.

## Methods

Al-Cu-Li model alloy samples were prepared from a high purity binary Al- 9 at.% Li alloy purchased from *Hauner Metallische Werkstoffe, Germany* as well as elemental Al and Cu with a purity of ≥99.9 % and ≥99.99 %, respectively. The samples were produced via arc melting with subsequent induction melting/tilt casting in high purity Argon atmosphere. Afterwards, a 70 minutes solution heat treatment at 788 K was applied on the bulk alloys in a nitrate salt melt. After solution heat treatment, samples were quenched in room temperature brine.

Samples for DSC measurements were subsequently naturally aged for at least 2 days at room temperature. Beforehand, it was confirmed by *in-situ* electrical resistivity measurements that after 2 days the natural aging process is completed in the studied alloy. Afterwards, artificial aging treatments at 433 K were performed in an oil bath. Samples for TEM and APT were prepared by cutting 300 *μm* thick sheets from the solution heat-treated bulk alloys using a low speed diamond saw. In order to remove potential stresses introduced during sample preparation, the specimens were afterwards subjected to a second heat treatment for 20 minutes at 803 K in an evacuated quartz ampule with subsequent quenching in room temperature brine and naturally aging at room temperature. As for the DSC samples, artificial aging treatments were performed in an oil bath at 433 K.

During sample preparation and solution heat treatment, the samples inevitably suffer from Li loss, making it necessary to determine the actual composition of the samples prior to further experiments. The compositions of the bulk and thin sheet samples were determined after solution heat treatment using Inductively Coupled Plasma Optical Emission Spectroscopy (ICP-OES). The different low temperature heat treatments applied did not result in changes in the sample compositions. In total, two batches of the same alloy were prepared with a determined composition of the alloy of 5.9 ± 0.25 at.% Li and 1.0 ± 0.05 at.% Cu. The composition of the model alloy was chosen in analogy to the technical Al-Cu-Li alloys 2099 and 2199 developed by *Alcoa Inc*.

For the TEM measurements, disc-shaped samples were punched from the heat-treated sheets and electropolished in a twin jet apparatus using a 2:1 methanol:nitric acid solution at 253 K operating at a voltage of 15 V. Prior to the measurement, samples were plasma cleaned. Conventional TEM measurements were performed on a Philips CM 12 at 100 kV and a FEI Titan 80–300 at an acceleration voltage of 300 kV. HRTEM and HAADF-STEM images were obtained on a FEI Titan 80–300 with a point resolution stated as 50.2 pixels/nm in the conventional mode and 56.9 pixels/nm in the STEM mode. The quantitatively analyzed diffraction pattern in this work was recorded on an image plate, which due to its high dynamic range and linearity allows for such a procedure^38^.

Samples for APT were prepared by wire saw cutting and subsequent electropolishing in a 2:1 methanol:nitric acid solution. The applied voltage in the electropolishing process was 5–8 V. APT measurements were performed on a CAMECA LEAP 4000 HR. All measurements were performed at a temperature of 25 K in voltage mode applying 5–14 kV, which has previously been reported to be a reasonable parameter set for Al-Li alloys^39^. The pulse fraction was set to 20–22.5% with a pulse frequency of 100 kHz and an evaporation rate of 0.002–0.006 atoms per pulse.

For this study, in total 7 Al-Cu-Li samples have been successfully analyzed with APT, yielding approximately 40 million reconstructed atoms overall. The alloy composition retrieved from all APT analyses was 5.68 ± 0.48 at.% Li and 0.96 ± 0.24 at.% Cu (standard deviations of the different reconstructions), showing a satisfactory agreement with the values obtained from ICP-OES. The 7 successful APT analyses belong to 4 different aging conditions: naturally aged, 5 minutes artificially aged at 433 K, 30 minutes artificially aged at 433 K and 60 minutes artificially aged at 433 K with 1.0 million, 30 million, 5.5 million and 2.7 million reconstructed atoms, respectively.

DSC measurements were performed on a Netzsch DSC 204 F calibrated over the entire temperature range. The measurements were performed with sealed and pierced Al crucibles. All samples were polished to ensure an optimal thermal contact to the crucible. The DSC measurements were performed at a heating rate of 10 K/min under Nitrogen atmosphere.

## Electronic supplementary material

Supplementary Information
