# Supplementary material for: L12 ordering and δ′ precipitation in Al-Cu-Li
Source: Sci Rep. 2017 Jun 12;7:3254. doi: 10.1038/s41598-017-03203-z (PMC5468256; doi:10.1038/s41598-017-03203-z)
Supplement: Supplementary file 1 — Supplementary Information [file 41598_2017_3203_MOESM1_ESM.pdf]

Supplementary Information: L1_2 ordering and δ' precipitation in Al-Cu-Li

Pascal Neibecker[1,2,3*], Michael Leitner[1], Muna Kushaim[2,4], Torben Boll[5], Dalaver Anjum[6], Tala'at Al-Kassab[2], and Ferdinand Haider[3]

[1]Heinz Maier-Leibnitz Zentrum (MLZ), Technische Universität München, Lichtenbergstr. 1, 85748 Garching, Germany
[2]King Abdullah University for Science and Technology (KAUST), 23955-6900 Thuwal, Kingdom of Saudi Arabia
[3]University of Augsburg, Department of Physics, Universitätsstr. 1, 86159 Augsburg, Germany
[4]Taibah University, Faculty of Science, Department of Physics, PO Box 344, Al-Madinah, Kingdom of Saudi Arabia
[5]Karlsruher Institut für Technologie (KIT), Institut für Angewandte Materialien - Werkstoffkunde (IAM-WK), Hermann-von-Helmholtz-Platz 1, 76344 Eggenstein-Leopoldshafen, Germany
[6]King Abdullah University for Science and Technology (KAUST), Imaging and Characterization Lab, 23955-6900 Thuwal, Kingdom of Saudi Arabia
[*]pascal.neibecker@frm2.tum.de

ABSTRACT

The Supplementary Information provides further Atom Probe Tomography reconstructions of the studied Al-1.0at.% Cu- 5.9at.% Li alloy. Additionally, in-depth information on the Atom Probe Tomography data analysis algorithm is given together with a detailed description of the Bragg-Williams-Gorsky (BWG) model implementation employed for the calculation of the Al-Li phase diagram in the main text.

Results

This section provides Atom Probe Tomography (APT) reconstructions excluded from the main text due to reasons of concision.

Fig. 1 shows the entire analysis volume of the 5 minutes at 433 K artificially aged sample, a section of which is presented in Fig. 5 of the main text. As in Fig. 5 of the main text, the central atoms of any 100 atom cell are depicted which are given a color depending on the Li concentration of the respective cell. Bright yellow stands for analysis cells with 8 at.% Li, dark red indicates a Li composition of $\geq$ 20 at.% Li. Clearly, the 5 minutes aged sample shows a homogeneous Li concentration without precipitates.

Fig. 2 shows the spatial distribution of Li in the 30 minutes at 433 K artificially aged condition. Here, few but well-defined spherical δ' precipitates with an average diameter of approximately 1.5 nm are visible in the sample. These findings convincingly support the findings of the binomial distribution analysis where for this sample/aging condition a second maximum at 22 at.% Li was observed in the spectrum.

Methods

Statistical analysis of decomposition processes with APT data

APT is a valuable tool for analyzing real space distributions of atoms and hence following chemical decomposition processes. This is especially interesting for analyzing atomic clustering in supersaturated solid solutions as it is the case in Al alloys undergoing a precipitation process under a specified annealing treatment. While the access to real space atomic arrangements basically allows for a straightforward data analysis for large-scale structures, the limited resolution of the atom probe of 0.5 nm in x-y direction as well as the limited detection efficiency of approximately 30 % make an elaborate statistical analysis of the reconstructed data necessary when analyzing small-scale clustering processes. Much effort has been put into designing algorithms allowing for identifying as small as possible cluster

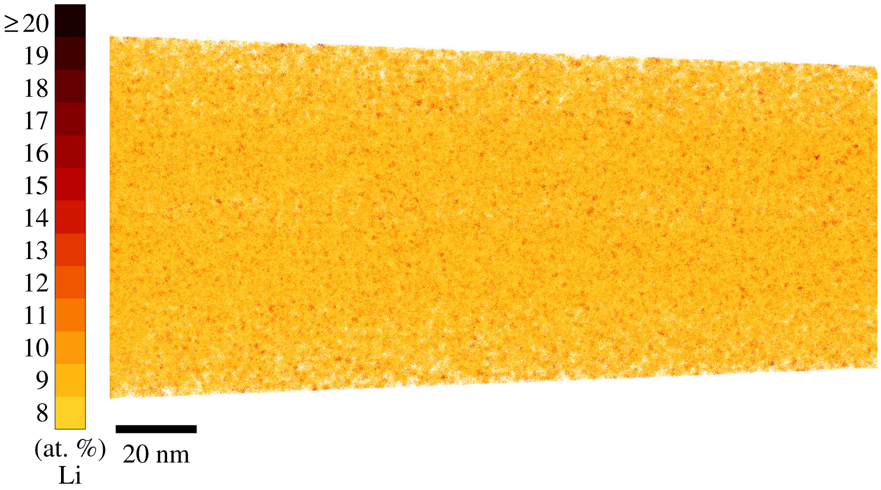

Figure 1. Visual representation of the spatial distribution of Li clusters in a 5 minutes at 433 K artificially aged sample showing a homogeneous Li distribution.

sizes of a specific element in a matrix[1–4]. Depending on the task to be accomplished, different approaches have proven to be successful.

In this manuscript, we are employing an algorithm identifying compositional variations in small, compact analysis volumes comprising M reconstructed atoms. Such a procedure has the advantage compared to more complex algorithms as e.g. described in Refs. 3, 4 that it is efficient for large data sets, weakly dependent on the chosen parameters in the APT reconstruction algorithm[2] and hence robust and reliable. Our choice of $M = 100$ constitutes an acceptable compromise between statistical significance (high at large M) and resolution for small-scale features (best at small M).[5]

Our algorithm first identifies the $M-1$ nearest neighbors of any of the N reconstructed atoms. This is done in an efficient way by first assigning all reconstructed atoms to boxes on a three-dimensional grid, giving $O(N)$ space and time complexity. Then, for a given central atom, a list of neighboring atoms is generated from the boxes in the vicinity, the distances from the central atom to all those atoms are computed, and the Mth smallest such distance d^* is determined by Hoare's quickselect algorithm.[6] If this cut-off distance d^* is larger than the minimal distance to any neighboring box that was not considered, this step is repeated with a larger set of included boxes. As the reconstructed densities are practically uniform, this happens only rarely, and the N spherical analysis cells of M atoms each can be found with an average time complexity of $O(MN)$. From this proximity classification, a two-dimensional histogram (for the special case of a ternary system) of the elemental make-up in the analysis cells is computed, which can trivially be marginalized to one-dimensional histograms of any given element.

In a perfectly random data set, the probability to find k atoms of a given species in a given M atom analysis cell is given by the binomial distribution

$$p(k) = \binom{M}{k} \cdot c^k \cdot (1-c)^{M-k} \tag{1}$$

where c is the concentration of the given species in the reconstruction. Comparing the one-dimensional local composition histogram of a given element to this random distribution directly allows to draw qualitative distinctions: If the reconstructed snapshot of the sample corresponds to a nucleation and growth mechanism at a stage where the precipitates have reached a size of M atoms or larger, the histogram will show a pronounced excess at concentrations corresponding to the precipitates, and the dominant matrix component will be shifted towards lower concentrations than the single component in the random model. If, in contrast, spinodal composition should be active in the sample,

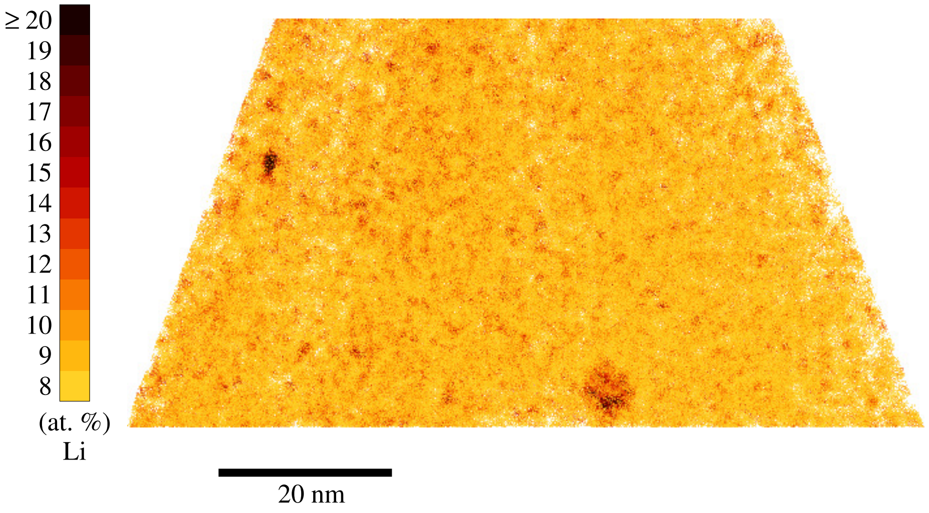

Figure 2. Visual representation of the spatial distribution of Li clusters in a 30 minutes at 433 K artificially aged sample showing the presence of spherical δ' precipitates

the histogram would display only a single component, which would not be shifted but broadened with respect to the random model, provided the analysis cell dimensions set by M is smaller than the critical length scale of spinodal decomposition. On the other hand, an ordered phase would have a local composition histogram that is narrowed compared to the random model due to the negative correlation of neighboring occupations in ordered phases. Yet, due to the limited spatial resolution of APT, a reliable observation of ordering processes is usually difficult.

The essence of binomial distribution analysis is to assign the reconstructed atoms to boxes and to sample the compositions of these boxes. Most published algorithms (e.g. Ref. 2) consider only a single choice of box boundaries, which corresponds to a significant loss of information, as the neighborhood relations crossing box boundaries are never used. In contrast, the intent of our algorithm is to use all available information for deducing spatial correlations by considering analysis cells centered around all reconstructed atoms. The drawback of our approach is that the analysis cells overlap, leading to correlations between the data points in the resulting histogram. As a consequence, the covariance matrix necessary to compute χ^2 is not diagonal any more and has a non-analytic form. However, we feel that this issue is not relevant, as the data size in typical APT reconstructions is so large that, in a purely statistical reasoning, the null hypothesis can be discarded practically always anyway.

Since our approach is based on a statistical analysis of the Li and Cu concentrations in the sample, artifacts of APT with respect to the reconstructed density have to be carefully accounted for. As already described in Refs. 7 and 8, irregular specimen geometries, as commonly present in crystalline samples, will cause trajectory aberrations that finally lead to unphysical local density distributions such as visible crystallographic poles and zone lines. Combined with element-specific field evaporation properties, especially in regions of irregular geometry, those poles and zone axes would bias any statistical analysis by pretending artificial concentration fluctuations that would erroneously be identified by the algorithm as decomposition processes. Hence, these anomalies have to be removed from the analysis volume. Further sources of artifacts such as the representation of blind spots of the multichannel plates in the reconstructed data, as well as detector efficiencies dependent on the applied voltage, especially for light elements such as Li, have to be checked for but were no major concern in the data sets obtained in this study. When excluding regions from the analysis volume, much caution has to be applied in order to find a reasonable trade-off between making use of as large as possible data sets and reducing efficiently the described artifacts. In our work, we used for this purpose a statistical method with visual feedback. Namely, we identified the reconstructed atomic density distribution by calculating the volume of the 100 nearest neighbors cells for every atom throughout the sample. In a

second step, this density distribution was depicted and crystallographic poles were efficiently identified on this basis and manually excluded.

The Bragg-Williams-Gorsky (BWG) model applied to the case of δ' precipitation in the Al-(Cu)-Li system

The Bragg-Williams-Gorsky (BWG) model as a mean field approach can be employed to describe qualitatively the thermodynamic situation with respect to the ordering-decomposition interdependencies in the pseudobinary Al-(Cu)-Li system. In the paper we present a reparametrized metastable phase diagram of the Al-(Cu)-Li system calculated with the BWG model as function of composition and temperature. The BWG model was firstly applied to this system by Khatchaturyan et al.[9] Yet, the plenitude of experimental data today allows for a more accurate parameterization of the model than the single data set used in the Khatchaturyan paper.

In the BWG model, the pseudobinary Al-(Cu)-Li system has two possible phases, i.e., the disordered solid solution and the ordered $L1_2$ phase. The free energy $F = U - T \cdot S$ of the ordered phase is then described via the following terms for the internal energy U and the entropy S:

$$U = 2 \cdot A \cdot (c_2 + c_1) \cdot c_2 + B \cdot ({c_1}^2 + 3 \cdot {c_2}^2) \tag{2}$$

$$S = -k_\mathrm{B} \cdot [c_1 \cdot \log(c_1) + (1 - c_1) \cdot \log(1 - c_1) + 3 \cdot c_2 \cdot \log(c_2) + 3 \cdot (1 - c_2) \cdot \log(1 - c_2)] \tag{3}$$

Here, the entropy S is the ideal entropy of mixing of one sub-lattice with Li concentration c_1 and three sublattices with concentration c_2, respectively, while A and B are free parameters of the model. The internal energy term U takes into account arbitrary pair interactions in a generic mean field approach. In this description, the expressions for the disordered solid solution follow from above expressions for the $L1_2$-phase by setting $c_1 = c_2 = c$. These expressions agree with the formalism in the original Khatchaturyan paper,[9] yet for the sake of a more intuitive access, we stick with the parameters c_1 and c_2 instead of rewriting them in terms of the order parameter.

To parametrize the model, a comprehensive set of experimental data was used, as outlined in the main text. Based on fitting these data sets, the free parameters of the model were determined as $A = 0.300\,\mathrm{eV}$ and $B = -0.092\,\mathrm{eV}$. Here we defined $A = \sum_{\vec{s} \in \Lambda_1} V(\vec{s})$, where $\vec{s} \in \Lambda_1$ are all vectors of generic type $\langle 0, \frac{1}{2}, \frac{1}{2} \rangle$ connecting sites on distinct sublattices and $B = \sum_{\vec{s} \in \Lambda_2} V(\vec{s})$, where $\vec{s} \in \Lambda_2$ are are all vectors of generic type $\langle 0, 0, 1 \rangle$ connecting sites on the same sublattice. $V(\vec{s}) = V^{\vec{s}}_{Al,Al} + V^{\vec{s}}_{Li,Li} - 2V^{\vec{s}}_{Al,Li}$ is the interaction constant for a connecting vector $\vec{s}$. Assuming the special case of only nearest neighbor (NN) and next nearest neighbor (NNN) interactions, this would correspond to respective interaction energies of $A/12 = 0.025\,\mathrm{eV}$ and $B/6 = -0.017\,\mathrm{eV}$. Note that Khachaturyan and coworkers' previous parametrization using a much smaller base of experimental data[9] corresponds to parameters of $A = 0.295\,\mathrm{eV}$ and $B = -0.0766\,\mathrm{eV}$.

References

1. Miller, M., Cerezo, A., Hetherington, M. G. & Smith, G. D. W. *Atom Probe Field Ion Microscopy* (Oxford University Press, New York, 1996).

2. Moody, M. P., Stephenson, L. T., Ceguerra, A. V. & Ringer, S. P. Quantitative Binomial Distribution Analyses of Nanoscale Like-Solute Atom Clustering and Segregation in Atom Probe Tomography Data. *Microsc. Res. Techniq.* **71**, 542–550 (2008).

3. Lefebvre, W., Philippe, T. & Vurpillot, F. Application of Delaunay tessellation for the characterization of solute-rich clusters in atom probe tomography. *Ultramicroscopy* **111**, 200–206 (2011).

4. Felfer, P., Ceguerra, A. V., Ringer, S. P. & Cairney, J. M. Detecting and extracting clusters in atom probe data: A simple, automated method using Voronoi cells. *Ultramicroscopy* **150**, 30–36 (2015).

5. Moody, M. P., Stephenson, L. T., Liddicoat, P. V. & Ringer, S. P. Contingency Table Techniques for Three Dimensional Atom Probe Tomography. *Microsc. Res. Techniq.* **70**, 258–268 (2007).

6. Hoare, C. A. R. Algorithm 65: Find. *Commun. ACM* **4**, 321–322 (1961).

7. Stephenson, L. T., Moody, M. P., Liddicoat, P. V. & Ringer, S. P. New Techniques for the Analysis of Fine-Scaled Clustering Phenomena within Atom Probe Tomography (APT) Data. *Microsc. Microanal.* **13** (2007).

8. Vurpillot, F., Cerezo, A., Blavette, D. & Larson, D. J. Modeling Image Distortions in 3DAP. *Microsc. Microanal.* **10**, 384–390 (2004).

9. Khachaturyan, A. G., Lindsey, T. F. & Morris, J. W. Theoretical Investigation of the Precipitation of δ' in Al–Li. *Metall. Trans. A* **19**, 249–258 (1988).
